# Spherical nucleic acids for precision medicine

**DOI:** 10.18632/oncotarget.1757

**Published:** 2013-12-31

**Authors:** Chad A. Mirkin, Alexander H. Stegh

**Affiliations:** Department of Chemistry, International Institute for Nanotechnology, Northwestern University, Evanston, IL; Ken and Ruth Davee Department of Neurology, The Northwestern Brain Tumor Institute, The Robert H. Lurie Comprehensive Cancer Center, International Institute for Nanotechnology, Northwestern University, Chicago, IL

With the advent of functional cancer genomics, precision medicine has begun to enter clinical practice. Therapeutic regimens are informed by the tumor's genotype to specifically target critical genetic aberrations that are the driving forces of disease progression. While several targeted small molecule inhibitors and biotherapeutic antibodies have been FDA-approved, in particular those targeting a cancer cell's kinome, it has become apparent that the majority of cancer genes represent unprecedented, non-enzymatic targets with unknown *modi operandi* that cooperate to drive cancer progression and therapy resistance. How can multiple, undruggable, and uncharacterized genes be therapeutically targeted? RNA interference (RNAi) comes to mind, but due to difficulties in delivering small interfering (si) or small hairpin (sh)RNAs robustly and safely to tumor sites, the delivery of oligonucleotide payloads represents a significant challenge and an unmet clinical need.

**Figure 1:**
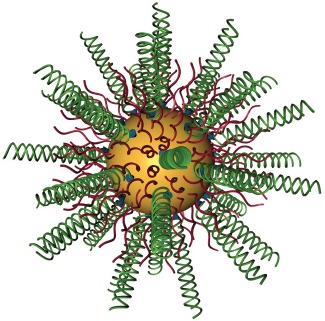
Spherical nucleic acids nanoconjugates Gold nanoparticles with a diameter of 13 nm are conjugated with RNA duplexes (green) containing an ethylene glycol spacer and a propylthiol group. Oligoethylene glycol (OEG)-thiol or poly(ethylene glycol) (PEG)-thiol (red) are used as passivation agents.

Nanotechnology continues to provide fundamentally different approaches to the treatment of genetic disease. In particular, spherical nucleic acids (SNAs), gold-based nanoconjugates functionalized with densely packed, highly oriented antisense DNA or siRNA oligonucleotides represent one of the most prominent and promising nanoscale gene regulation platforms [1]. Specifically, these nanoconjugates typically are comprised of a 13 nm gold nanoparticle core that is decorated with a corona of thiolated double-stranded RNAs (Figure 1). SNAs show robust cellular uptake via scavenger-receptor-dependent endocytosis without the use of toxic, auxiliary transfection agents or viral delivery platforms [1] and exhibit extraordinary stability in physiological environments and robust resistance to nuclease degradation [1, 2].

To evaluate SNAs as a platform for biotherapeutic gene silencing in cancer, we synthesized SNAs targeted to the oncogene Bcl2-Like12 (Bcl2L12), which is over-expressed in several cancer types, most prominently in Glioblastoma multiforme (GBM), the most aggressive and prevalent manifestation of malignant brain tumors. Bcl2L12 is a an atypical member of the Bcl-2 protein family, characterized by proline-rich and a *C*-terminal 14 amino acid sequence with homology to the BH (Bcl-2 Homology) 2 domain found in several members of the Bcl-2 family [3, 4]. Bcl2L12 is overepxressed in >90% of human primary GBM specimens and exhibits low or undetectable levels in cells of glial origin in the normal brain surrounding tumor tissue or in low-grade astrocytoma [4]. Analysis of 343 glioma patients in the Repository of Molecular Brain Neoplasia Data (REMBRANDT) identified *Bcl2L12* as a potential prognostic factor, as GBM patients with high-level overexpression of *Bcl2L12* mRNA have shorter progression-free survival compared to patients with low expression or underexpression of *Bcl2L12* (*p*<0.001). On the cellular level, enforced expression of Bcl2L12 confers marked apoptosis resistance, induces malignant transformation, and promotes high-grade glioma progression *in vivo* [4]. Mechanistically, Bcl2L12 inhibits apoptosis by neutralizing effector caspases [4]. Bcl2L12 physically interacts with caspase-7, blocks proteolytical processing by upstream caspases, and induces transcriptional upregulation of the small heat shock protein αB-crystallin, which directly binds to and inhibits caspase-3. In the cell nucleus, Bcl2L12 interacts with the p53 tumor suppressor. Consequently, Bcl2L12 expression antagonizes replicative senescence without concomitant loss of p53 or p19*Arf*, blocks p53-dependent apoptosis, impedes the capacity of p53 to bind to target gene promoters and to transcriptionally induce target mRNA expression, e.g. p21 [5]. Correspondingly, copy number and mRNA profiles obtained from The Cancer Genome Atlas (TCGA), together with protein analyses of human GBM specimens, showed significantly lower Bcl2L12 expression in the setting of genetic p53 pathway inactivation [5]. Thus, the multi-functional Bcl2L12 protein is an important oncoprotein and prognostication factor in GBM.

To neutralize Bcl2L12 expression in GBM cells and tumors, we synthesized SNAs targeted to Bcl2L12 (siL12-SNAs) [6]. We identified conjugates that were capable of reducing Bcl2L12 protein abundance by 60-95% in patient-derived tumor neurospheres (TNS) and transformed glioma cells. As shown with Bcl2L12-targeting siRNA and shRNAs [4, 5, 7-9], SNA-mediated knockdown of Bcl2L12 resulted in enhanced effector caspase and p53 activation, confirming the functionality of SNA-mediated knockdown of Bcl2L12. Using 5'-RNA-ligand-mediated-*Rapid Amplification of cDNA Ends (RACE)*, we positively identified the mRNA cleavage product that resulted from siL12-SNA-triggered, RNA-induced silencing complex (RISC)-mediated RNAi. Building on robust cellular uptake and knockdown triggered by SNAs, can we safely and effectively deliver SNAs to intracranial tumor sites?

In vivo Imaging System analysis of mice intravenously injected with fluorochrome-tagged SNAs revealed that SNAs cross the blood-brain and blood-tumor barriers, and preferentially accumulated in intracerebral glioma elements [6]. Selective intratumoral accumulation is likely due to the Enhanced Permeability and Retention effect, i.e., the increased accumulation of nanomaterials in tumors due to abnormal form and architecture of tumor blood vessels. Biodistribution analysis revealed that up to 1% of the total amount of SNAs injected was found within the tumor, with the majority of SNAs accumulating in the liver and spleen [6]. Accumulation and pervasive dissemination into extravascular tumor parenchyma translated into robust intratumoral protein knockdown, increased intratumoral apoptosis, and impaired tumorigenicity as measured by reduced tumor burden. In the absence of systemic toxicity, siL12-SNAs prolonged survival of TNS-derived xenogeneic mice [6]. Thus, SNAs represent a powerful platform for systemic RNAi-mediated biotherapeutic gene silencing for the treatment of GBM. The presence of gold and the ability to co-functionalize nucleic acids with imaging agents, e.g., Gadolinium, allows for the determination of the intratumoral concentrations of SNAs by mass spectrometry and the tracking of SNAs via a variety of imaging techniques, respectively.

Bcl2L12 is a universal factor that regulates apoptosis in response to a broad spectrum of chemo-and targeted therapies. To further increase anti-GBM activity of Bcl2L12-targeting SNAs, it will be instrumental to develop combinatorial treatment regimes, particularly those inhibiting critical driving oncogenes such as (receptor) tyrosine kinases and downstream PI3K-Akt and Ras-MAPK signaling axes. Upon evaluation of safety in non-human primates, clinical trials in humans aim to determine whether SNAs targeted to Bcl2L12 can cross the blood-brain barrier, accumulate in and disseminate throughout tumors, and trigger robust knockdown of Bcl2L12. Finally, we envision utilizing SNAs as a therapeutic modality for other malignancies that are characterized by overexpression of Bcl2L12, such as skin and breast carcinomas, and leveraging the ability of SNAs to cross the blood-brain barrier for the treatment of other central nervous system diseases.
